# Prevalence of integron classes in Gram-negative clinical isolated bacteria in Iran: a systematic review and meta-analysis

**DOI:** 10.22038/ijbms.2018.32052.7697

**Published:** 2019-02

**Authors:** Ali Pormohammad, Ramin Pouriran, Hadi Azimi, Mehdi Goudarzi

**Affiliations:** 1 Student Research Committee, Department of Microbiology, School of Medicine, Shahid Beheshti University of Medical Sciences, Tehran, Iran; 2 School of Medicine, Shahid Beheshti University of Medical Sciences, Tehran, Iran; 3 English Language Teaching Department, School of Medicine, Shahid Beheshti University of Medical Sciences, Tehran, Iran; 4 Department of Microbiology, School of Medicine, Shahid Beheshti University of Medical Sciences, Tehran, Iran

**Keywords:** Bacteria, Integron, Iran, MDR, Meta-Analysis

## Abstract

**Objective(s):**

Integrons, as a potential element in the distribution and maintenance of drug resistance, have thoroughly been established. It is known that the high prevalence of integrons in multidrug-resistant (MDR) clinical isolates has become a serious public health concern. The objective of the present study was to determine the frequency of different classes of integrons in clinical isolates in Iran.

**Materials and Methods:**

Electronic global databases were systematically searched. The raw data for integrons among bacterial isolates were collected and their prevalence was analyzed using Comprehensive Meta-Analysis V2.0 (Biostat, Englewood, NJ, USA) software.

**Results:**

In a comprehensive literature review, 29 eligible studies were determined with their meta-analyses indicating the prevalence of integron class 1 to be 41% (95% CI 36.3-46.1) and integron class 2 as 17.7% (95% CI 13-23.3) in Gram-negative bacteria. The highest prevalence of integron class 1 was reported in *Acinetobacter spp* (58%) while the highest prevalence of integron class 2 was reported in *Shigella* isolates (83.7%). The frequencies of class 1 integron in MDR (79%) and non-MDR isolates (41%) were higher than those for class 2 integron in MDR (13.4%) and non-MDR isolates (17.7%).

**Conclusion:**

The current systematic review demonstrated the significant presence of integrons among clinical isolates. Our analysis showed that measures such as estimates of the prevalence of this transposable element and diligence in continued surveillance might be necessary to prevent its spread.

## Introduction

Antimicrobial resistance, as a growing threat, is the cause of 700’000 deaths worldwide and is forecasted to cause 10 million deaths a year by 2050 in the absence of coherent programs to combat it. These increasing threats will perhaps grow even more dramatically in the developing countries (1). According to available data, antimicrobial resistance is linked to occurrence and distribution of genetic elements (2). The genetic elements were primarily described in the late 1980s; apparently, they have been extensively recognized for their transpicuous role for the spread of resistance determinants distinctly among Gram-negative strains. Obviously, integrons as a peculiar group of genetic elements, have general and important roles in bacterial adaptation and genome evolution (3). Recently, integrons, as a common component of bacterial genomes, are widely known for their role in the dissemination of antibiotic resistance (4). Integrons form a complex mobilome in the majority of environments and, in addition, they are capable of moving between species over evolutionary periods, and have a vast pool of new genes available whose functions are not still transparent (5, 6). In fact, integrons contain three essential core features: 1) integrase, a member of the tyrosine recombinase family, encoded by *intI*, which catalyzes recombination of captured gene cassettes, 2) a primary integron-associated recombination site, *attI*, and 3) an integron-associated promoter, *Pc*, which lies between *attI* site and *intI* gene (4, 7, 8).

Although integrons are not mobile in their own right, they are considered as major players in the development and spread of antimicrobial resistance, particularly among Gram-negative bacteria (9). There are five classes of “mobile” antibiotic resistance-associated integrons. Classes 1, 2, and 3 are frequently detected from clinical sources; class 4 is primarily detected on the SXT element of *Vibrio cholera*, and finally, class 5, which is identified on the pRSV1 plasmid in *Alivibrio salmonicida *(10-12). Antibiotic resistance integrons have numerous characteristics which are common among them. For instance, they are ordinarily mobile and their cassettes sequence is short and prevalently encoded for antibiotic resistance (13). Contemporary, the antibiotic resistance phenomenon has dramatically been increased in antibiotic resistance-associated integrons in patients, thus consequently, increasing the contingency of new and more complex resistance to abundant antibiotic classes, heavy metals, and disinfectants among bacterial strains (14). Conversely, it was demonstrated that three classes of mobile integrons, including class 1, 2, and 3, are involved in the multi-drug resistance phenotypes. The mentioned integrons provide pathogens with a gene capture system which improves the challenges for multiple-antibiotic treatment regime (15).

Unfortunately antimicrobial screening programs have not received enough attention in Iran and currently infections caused by multidrug-resistant bacterial strains are among the main factors influencing morbidity and mortality in Iranian patients (16). The importance of antibiotic resistance-associated integrons in clinical settings has notably been reflected in their global epidemiological surveillance, monitoring, prevalence, and evolution. Apparently, some reports present the significance between multidrug resistance and integron carriage among clinical isolates fermentative and non-fermentative Gram-negative bacilli in Iran (17-19); nevertheless, there is insufficient information regarding the structure and epidemiology of antibiotic resistance-associated integrons among bacterial populations isolated in clinical samples in Iran. Therefore, the purpose of the present meta-analysis was to confirm the prevalence of antibiotic resistance-associated integrons class 1 and 2 among the clinical bacterial isolates in published reports in Iran. 

## Materials and Methods

***Data acquisition***

A literature search of the English-language databases including MEDLINE, Web of Science, Scopus, Embase, and Science Direct was conducted on the studies published from Jan 1, 2000 to Jan 31, 2016. In addition, the entire relevant articles in national databases such as Iranmedex (www.iranmedex.com), Scientific Information Database (www.sid.ir), Magiran (www.Magiran.com), Irandoc (www.irandoc.ac.ir), and Iranian National Library (www.nlai.ir) were searched using a similar strategy and related Persian keywords. The search was restricted to original research articles. The Medical Subject Headings (MeSH) keywords and synonyms used included “integrons”, “integron classes”, “chromosomal integrons”, “gene cassette”, “mobile genetic elements“, “antibiotic resistance”, “bacteria”, “drug resistant”, “multidrug resistant”, “prevalence”, and “Iran”. In addition, we searched related journals, citations lists (backward citation), and references (forward citation) and corresponded with authors (recommended with Cochrane guideline) (20). Furthermore, no contact was made with the expert authors regarding our previous experiences (21, 22). To improve the sensitivity and specificity, the literature review was carried out by three independent investigators. The present study was conducted according to the systematic review following PRISMA guidelines (23).

**Figure 1.**
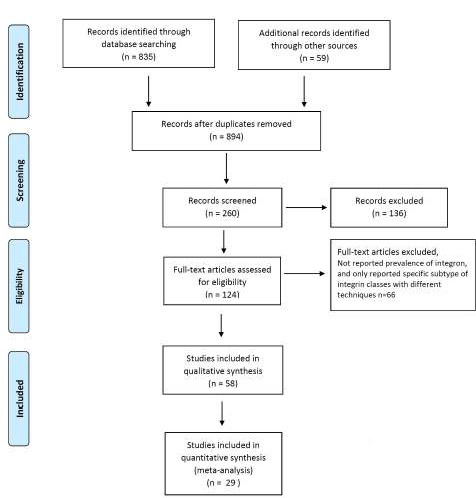
Flow diagram of literature search and study selection

**Figure 2:**
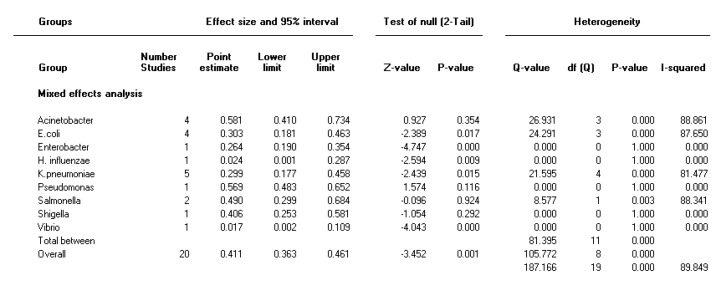
Forest plot of the meta-analysis on prevalence of integron class 1 in Gram-negative bacteria

**Figure 3:**
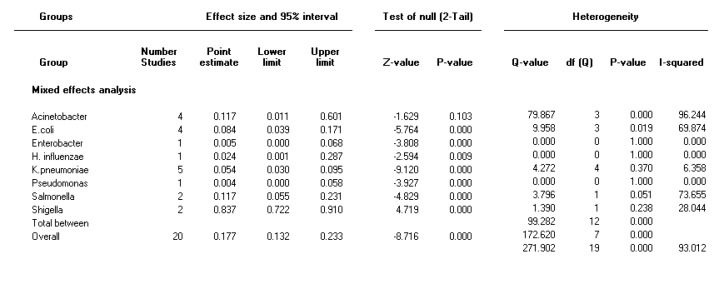
Forest plot of the meta-analysis on prevalence of integron class 2 in Gram-negative bacteria

**Figure 4:**
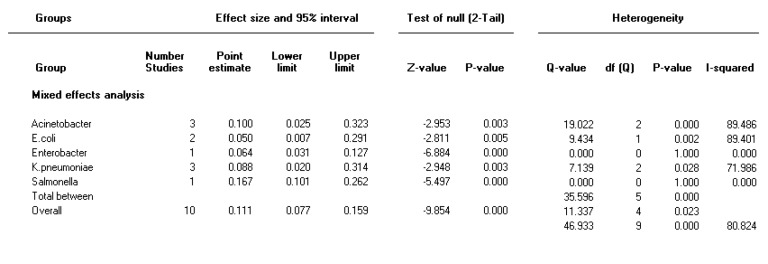
Forest plot of the meta-analysis on prevalence of both integron classes 1 and 2 in Gram-negative bacteria

**Figure 5:**
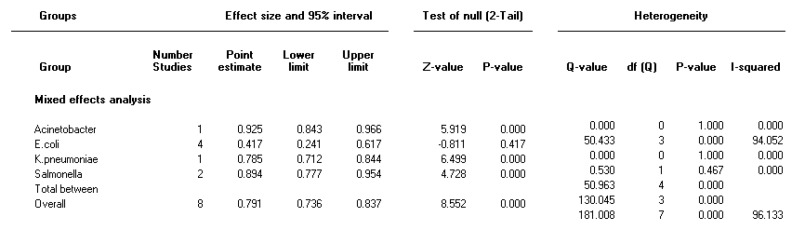
Forest plot of the meta-analysis on prevalence of integron class 1 in Gram-negative multi-drug resistance bacteria

**Figure 6:**
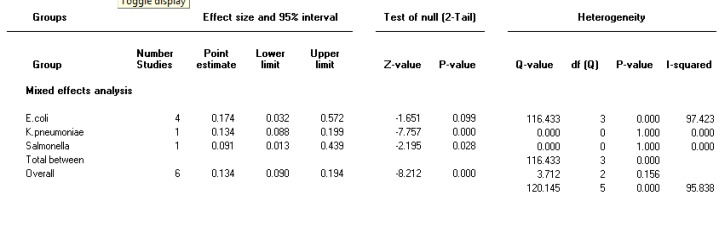
Forest plot of the meta-analysis on prevalence of integron class 2 in Gram-negative MDR bacteria

**Figure 7:**
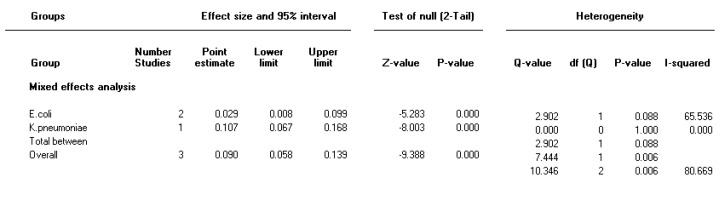
Forest plot of the meta-analysis on prevalence of both integron classes 1 and 2 in gram-negative multi-drug resistance bacteria

**Table 1:** Characteristics of studies included in the meta-analysis

**First author**	Published	Province	No. Isolate bacteria	Organism	Detection method	No. Int1	No. Int2	No. Both
**Ranjbar** **(** 25 **)**	2007	Tehran	57	*Shigella sonnei *	PFGE	UN	50	UN
**Japoni(** 26 **)**	2011	Shiraz	88	*Acinetobacter*	RFLP	42	3	2
**Adabi(** 27 **)**	2009	Tehran, Zahedan, Golestan, and Qom	60	*Vibrio cholerae*	PCR	1	UN	UN
**Taherikhani** **(** 28 **)**	2011	Tehran	100	* Acinetobacter baumannii*	Repetitive element palindromic PCR	58	14	9
**Peymani (** 29 **)**	2012	Tabriz	100	*A. baumannii*	UN	80	0	UN
**Naghoni (** 30 **)**	2010	Tehran	138	*Salmonella spp*	PCR	54	11	UN
**Firoozeh (** 31 **)**	2011	Tehran	58	*Salmonella spp*	PCR	UN	UN	UN
**Rezayi (** 32 **)**	2011	Tabriz	140	*Escherichia coli*	PCR	UN	UN	UN
**Mirnejad (** 33 **)**	2013	Tehran	50	*A. baumannii*	PCR	21	41	15
**Rajaei (** 34 **)**	2011	Tehran	84	*Salmonella*	UN	50	14	14
**Mobarak (** 35 **)**	2013	Tehran	104	*Klebsiella pneumoniae*	PCR	22	3	UN
**Derakhshan (** 36 **)**	2014	Tehran	31	*K. pneumoniae*	PCR	8	0	UN
**Eftekhari (** 37 **)**	2013	Tehran and Khorasan	32	*Shigella spp*	PFGE	13	25	UN
**Kargar (** 38 **)**	2014	Yasouj	164	*E. coli*	PCR	UN	UN	UN
**Bromand (** 39 **)**	2015	Tehran	20	*Haemophilus influenzae*	PCR	0	0	UN
**Peerayeh (** 40 **)**	2015	Tehran	123	*A. baumannii*	MLVA	UN	UN	UN
**Haddadi (** 41 **)**	2015	Karaj	111	*E. coli*	PCR-RFLP	25	1	UN
**Memariani** ** (** 42 **)**	2014	Tehran	42	*E.coli*	PCR	24	2	UN
**Salimian (** 43 **)**	2015	Tehran	110	*Enterobacter spp.*	PCR	29	0	7
**Azami (** 44 **)**	2013	Tehran	130	*Pseudomonas aeruginosa*	PCR	74	0	UN
**Ashayeri (** 45 **)**	2014	Tehran	35	*K. pneumoniae*	PCR	21	3	2
**Shams (** 46 **)**	2015	Tabriz	72	*E. coli*	UN	11	11	9
**Shams (** 46 **)**	2015	Tabriz	63	*K. pneumoniae *	PCR	22	5	14
**Rezayi ** **(** 47 **)**	2012	Tabriz	150	*K. pneumoniae*	PCR	UN	UN	UN
**Seyedjavadi (** 48 **)**	2013	Tehran	174	*E. coli*	PCR	59	22	3
**Seyedjavadi (** 48 **)**	2013	Tehran	30	*K. pneumoniae*	PCR	4	0	0
**Japoni (** 49 **)**	2008	Shiraz	200	*E. coli*	PCR-RFLP	UN	UN	UN
**Fallah (** 50 **)**	2012	Tehran	200	*E. coli*	RFLP	UN	UN	UN

* UN=UN know

**Table 2:** Meta-analysis, prevalence of integron class 1 and 2 in all clinical and MDR isolates

**Bacteria**	**integron classes**	**In all isolates**		**In MDR isolates**	
		Prevalence (%)	Heterogeneity test, I2 (%) (P value)	Prevalence (%)	Heterogeneiy test, I2 (%) (P value)
***E. coli***	Int 1	119/399 (30.3)	87.64 (0)	165/389 (49.3)	94.63 (0)
	Int 2	36/399 (8.4)	69.87 (0.019)	84/389 (24.7)	98.01 (0)
***K.*** ***pneumonia***	Int 1	77/263 (29.9)	81.47 (0)	77/149 (51.7)	0 (0)
	Int 2	11/263 (5.4)	6.35 (0.37)	20/149 (13.4)	0 (1)
***Acinetobacter spp***	Int 1	201/338 (58.1)	88.86 (0)	103/110 (93.3)	0 (0.43)
	Int 2	58/338 (11.7)	96.24 (0)	13/30 (43.3)	0 (1)
***Salmonella spp***	Int 1	104/222 (49)	88.34 (0.003)	49/54 (89.4)	0 (0.46)
	Int 2	25/222 (11.7)	73.65 (0.051)	1/11 (9.1)	0 (1)
***Shigella spp***	Int 1	13/32 (40.6)	0 (1)	-	-
	Int 2	75/89 (83.7)	28.04 (0.23)	-	-
***V. cholera***	Int 1	1/60 (1.66)	0 (1)	-	-
***H. influenzae***	Int 1	0/20 (0)	0 (1)	-	-
	Int 2	0/20 (0)	0 (1)	-	-
***Enterobacter spp*** **.**	Int 1	29/110 (26.36)	0 (1)	-	-
	Int 2	0/110 (0)	0 (1)	-	-
***P. aeruginosa***	Int 1	74/130 (56.92)	0 (1)	-	-
	Int 2	0/130 (0)	0 (1)	-	-

***Inclusion and exclusion criteria***

Evaluation of the studies for inclusion in the current meta-analysis was done independently by two experts. Inclusions of the studies were conducted following three stages: titles, abstracts, and full-text evaluation. In all included articles, a standard molecular assay (polymerase chain reaction (PCR), restriction fragment length polymorphism (RFLP), pulsed-field gel electrophoresis (PFGE), multiple locus variable-number tandem repeat analysis (MLVA)) was performed for detection of integron class 1 and class 2 among clinical isolates of Gram-negative bacteria. Indeed, some studied were excluded from the analysis because of the following reasons: studies which included only specific groups of patients, those which identified integrons using different techniques, and those which did not report the prevalence of integrons. Moreover, reviews, case reports, and abstracts without appropriate data were also excluded.

***Quality assessment and Data extraction***

Full manuscripts of the included studies were assessed by three investigators. Disagreements in quality assessment were discussed and resolved by consensus. Quality assessment of obtained articles was performed according to the checklist which was provided by the Joanna Briggs Institute (24). For all studies, the extracted data included the following: first author’s name, data of carrying out the study, publication date, study location, methods for conducting studies, source of samples, sample size, prevalence of each integron class in all the isolates, and prevalence of each single integron class in multidrug-resistant (MDR) isolates. In addition, information on bacterial species, antibiotic resistance rate, and the strain type (if reported) were extracted from the included studies.

***Data pooling and statistical analysis***

The pooled prevalence of integron classes in different species of bacteria and MDR isolates were calculated for each bacterial species. Random effect model was used to pool the estimated effects. The analysis was carried out using Comprehensive Meta-Analysis Software Version 2.0 (Biostat, Englewood, NJ) and determination of heterogeneity among studies was undertaken making use of the chi-squared test (Cochran’s Q) to assess the appropriateness of pooling data. *I2* value, with *I2* ≥75% denoted a high degree of statistically significant heterogeneity. The point estimates of effect size, prevalence of integron classes, and its 95% confidence interval (95% CI) were estimated in each study. Values *P<*0.05 were considered as statistically significant.

## Results

***Characteristics of included studies***

Primarily, a total of 894 articles were collected (Figure 1). In the secondary screening, 770 articles were excluded based on the title and abstract evaluation. As a matter of fact, the exclusion were mainly because of the following reasons: the articles were based on case reports or reviews, assessment of typing methods was based on specific class of integrons, the samples were isolated from integrons from animals or environment, and reported integrons were from specific patients. In the next step, 66 of the remaining 124 studies were excluded upon a full text assessment because they reported specific subtypes of integron classes with different techniques. A total of 29 eligible studies were chosen for further investigation. Characteristics of the selected articles are presented in Table 1. As a matter of fact, the entire included studies were cross-sectional studies and the majority of the included studies detected intergron classes using PCR assay. It is worth noting that the bacteria were isolated from different clinical samples including blood, urine, cerebrospinal fluid (CSF), Broncho Alveolar lavage (BAL), and other body fluids.

***The prevalence of integron in different species of bacteria***

The heterogeneity test indicated that there were heterogeneities between studies for integron class 1 (I2=89.8, *P<*0.001) and for integron class 2 (I2=93, *P<* 0.001); therefore, the random effect model was used to combine the prevalence of integron class 1 and 2. As it is present in Figure 2 and 3, the combined prevalence of integron class 1 and integron class 2 were 41 % (95% CI 36.3-46.1) and 17.7% (95% CI 13-23.3), respectively, in gram-negative bacteria in Iran. Moreover, Figure 2 and 3 shows the forest plot of meta-analysis of integron class 1 and 2 prevalence in gram-negative bacteria, respectively.

***The prevalence of integron class 1 and integron class 2 in different species***

As shown in Table 2, the highest pooled prevalence rates across all reports for integron class 1 was 58% for *Acinetobacter spp *and the highest pooled prevalence for integron class 2 was 83.7% in *Shigella *isolates. In the 29 included studies, integron 3 was not detected, except in Kargar *et al.* (2014) study, which was 18/164 (10.97 %) in *Escherichia coli *isolates. Pooled prevalence rates of integron class 1 and 2 in Gram-negative bacteria with time point subgrouping are shown in Figure S1 and S2 (in supplementary materials).

***The prevalence of both class 1 and 2 integron in different species of bacteria***

The random effect model was used to combine the prevalence of both integron class 1 and 2 due to significant heterogeneity (I2=81, *P<*0.001). Pooled prevalence of both integron class 1 and 2 was 11 % (95% CI 7.7-16) in Gram-negative bacteria. Moreover, the highest and lowest pooled prevalence rates in integron class 1 and 2 were 16.7 % in *Shigella *and 5% in* E. coli *isolates, respectively (Figure 4). 

***The prevalence of integron class 1 in multidrug resistance isolates***

The heterogeneity test indicated that there were heterogeneities (I2=96, *P<*0.001) between studies; therefore, the random effect model was used to combine the prevalence of integron class 1 in MDR isolates. Pooled prevalence of integron class 1 was 79 % (95% CI 73.6-83.7) in Gram-negative MDR isolates. Moreover, the highest and lowest pooled prevalence in integron class 1 was 92.5 % in *Acinetobacter spp *and 41.7 % in* E. coli *isolates, respectively (Figure 5).

***The prevalence of integron class 2 in MDR isolates***

The heterogeneity test indicated that there were heterogeneities (I2=96, *P<*0.001) between studies; therefore, the random effect model was used to combine the prevalence of integron class 2 in MDR isolates. Pooled prevalence of integron class 2 was 13.4 % (95% CI 9-19.5) in gram-negative MDR isolates (Figure 6).

***The prevalence of both integron class 1 and 2 in multidrug resistance isolates***

The random effect model was used to combine the prevalence of both integron class 1 and 2 due to significant heterogeneity (I2=80, *P<*0.001). Pooled prevalence of both integron class 1 and 2 was 9 % (95% CI 5.8-14) in Gram-negative MDR isolates (Figure 7).

## Discussion

Recently, the spread of integron has become a dilemma for infection control in health care systems. The current systematic review focused on the prevalence of integrons in the isolates recovered from clinical samples and their interactions with MDR in Iran. Although different comprehensive analysis for bacterial genomes revealed that approximately 9-17% of sequenced bacterial genomes carry an integron integrase (51), the current systematic review reports the rates of 41% and 17.7% for the existence of integron class 1 and 2 among clinical strains in Iran. Based on our analysis, the prevalence of both class 1 and 2, simultaneously, in clinical isolates was found to be 11 %. The high prevalence of integron was detected among *Acinetobacter spp* isolates (58 %).

Given the high prevalence of integron class 1 in *Acinetobacter spp* isolates, several hypotheses can be deduced. First, improper use of antibiotic for treatment of *Acinetobacter spp* leads to express gene cassettes contained within integrons class 1 and, as a result, MDR will occur. Second, the ability of integrons to acquire new gene cassettes, and to rearrange those already within arrays, due to antibiotic selective pressure, leads to disseminating antibiotic resistance among *Acinetobacter *spp clinical isolates. Finally, failure to implement standard principles of infection control in hospitals and health care settings leads to survival of MDR *Acinetobacter *spp isolates carrying integron and dissemination of resistance integrons between other *Acinetobacter *spp isolates and bacteria. 

Although it is well established that in *Shigella* spp, the spread of resistance genes is mostly facilitated by the ability of this bacterium to acquire transposons or plasmids, the present analysis revealed that the highest prevalence of integron class 2 was 83.7 % in *Shigella *isolates. Unfortunately, in Iran, physicians treat patients with diarrhea without considering the susceptibility testing results and even in many cases patients with diarrhea take antibiotic therapy prior to visiting a doctor, regardless of whether the diarrhea was caused by bacteria or virus. Of course, the improper use of antibiotics in domestic animals either therapeutically or for the purpose of growth promotion which leads to MDR patterns and high occrance of mobile resistance integrons should not be overlooked (52). Therefore, as a part of the public health strategy, it is important to monitor the prevalence of integron and regional and local antimicrobial resistance profiles of* Shigella *clinical isolates.

Our analyses showed that the frequencies of class 1 integron in MDR (79%) and non MDR isolates (41%) were higher than those of class 2 integron in MDR (13.4%) and non MDR isolates (17.7%). Particularly, the high frequency of class 1 integron, as a major experimental model of integron; moreover, its role in the distribution and spread of antimicrobial resistance has been well established. It seems that the location of class 1 integrons on genetic elements such as conjugative plasmids and transposons provide further support of this idea that class 1 integrons are widespread as compared to the other classes (15). 

According to our analyses, only one study reported the existence of class 3 integron (10.97%), which is in accordance with world reports (53). Up to now, class 3 integrons have been described in *Acinetobacter *spp., *Alcaligenes*, *Citrobacter freundii*, *E. coli*, *K. pneumoniae*, *P. aeruginosa*, *P. putida*, *Salmonella *spp, and *Serratia marcescens*. Based on the previous published data, it is demonstrated that class 3 integrons from clinical contexts are associated with antibiotic resistance. Therefore, they do not carry a great diversity of gene cassettes (54). 

Our results clearly suggest that integron, as indicator of drug resistance, could pose a challenge for public health surveillance. Ostensibly, the emergence and increasingly widespread of introng-related resistance among clinical strains in Iran is a challenge for public health surveillance and support the hypothesis of improper use of antimicrobial agents, because of the low cost of many drugs, inappropriate antibiotic prescription protocols, and failure to implement standard principles of infection control. In this regard, physicians and patients should be educated about prescriptions and use of drugs.

As previously stated, antibiotic resistance, as a global multifaceted phenomenon, has become a major threat to global health which highlights the need for heightened awareness among clinicians, veterinarians, scientists, and policymakers and also implementation of action plans to reduce the spread of antimicrobial-resistant microorganisms (1). The increasing global phenomenon of antimicrobial resistance is commonly linked to the “selective pressure” caused by the inappropriate use, overuse, or underuse of antibiotics in humans and animals. On the other hand, the role of antibiotics usage in agriculture that leads to antibiotic resistance in bacteria living on plant surfaces, which might then be transferred into clinically important bacteria, should not be ignored (1, 2, 16).

Iran is a middle income country that consumes a high volume of antibiotics in the world. Overall, Iranian Health Ministry broadly outlines different policies as cornerstones of the effort to tackle antimicrobial resistance including 1) education and improvement of awareness about antimicrobial resistance and self-medication, 2) prohibition of antibiotic sales without a medical prescription, 3) establishment of national laboratories with the ability to identify resistant bacteria, 4) recruitment of clinical pharmacists as an important stakeholder beside the other physicians in respect to antibiotic management, and 5) implementation of national surveillance program and standard infection control measures to reduce the incidence of infection and limited and rational use of antimicrobial agents (55, 56).

The present study had some limitations which should be considered prior to interpretation of the results. Indeed, the present meta-analysis, included studies from almost all regions of Iran. In fact, only the chosen studies were included in the analysis; therefore, the number of eligible studies selected could possibly affect the statistical analysis for detecting funnel plot asymmetry, which could lead to publication bias. As a result, because of the restricted information obtained from the included articles, the demographic data, history of hospitalization, and previous antibiotic treatment history could not be analyzed. There was also a considerable heterogeneity among the included studies.

## Conclusion

Our data supports the claim that integrons are prevalent in Iran. The emergence of integron and extremely rapid spread of MDR in different bacteria species is becoming a serious public health concern in Iran. The present systematic review presents the prevalence of integrons in different bacteria species. Overall, the current article emphasizes that detection of integron as remarkable genetic platforms with the ability to acquire, rearrange, and express diverse genes should be prioritized in different bacteria species isolated from patients in Iran.
